# Beyond the role of participant: a firsthand account of the experiences of a patient-oriented research team

**DOI:** 10.1186/s40900-021-00323-9

**Published:** 2021-11-07

**Authors:** Lindsey Boechler, Steven Renwick, Abdullateef Alabi, Harold de la Torre, Susheel Kumar, Harmanpreet Singh, Roshan Xavier, Dalise Hector, Lauren McTaggart, Jennifer Shrubsole

**Affiliations:** 1 Centre for Health Research, Improvement and Scholarship, Saskatchewan Polytechnic, Moose Jaw, Canada; 2 Saskatchewan Polytechnic, Moose Jaw, Canada; 3 Patient Partner, Moose Jaw, Canada; 4 Patient Partner, Saskatoon, Canada; 5 Patient Partner, Regina, Canada; 6 Prairie Skies Integration Network, Moose Jaw, Canada; 7 Moose Jaw Multicultural Council, Moose Jaw, Canada; 8 Saskatchewan Polytechnic, Moose Jaw, Canada

**Keywords:** Patient-oriented research, Immigrant health, Health disparities, Accessible health care, Canada

## Abstract

**Background:**

Immigrants often find accessing and navigating the healthcare system difficult upon arriving in Canada. Existing challenges of accessing healthcare due to differing cultural norms, language barriers, limited health literacy, and system complexity have been outlined extensively in literature; however, evidence-informed practices to mitigate these disparities have yet to be determined.

Our research team took a patient-oriented research (POR) approach to learn more about the lived experiences of immigrants as they attempt to access and navigate the health system upon immigrating to Canada. POR is a method that involves patients beyond the role of participant, recognizing the lived experiences of patients as expertise and empowering patient partners to drive research priorities. This approach empowers patient partners to steer the direction of research, ensuring the study is relevant and patient priorities are addressed.

**Main body:**

In this article, we define POR and share our team’s experience of engaging in POR by providing a synopsis of team member recruitment, research priority establishment, and relationship building. We also share how joining forces with patient partners, rather than solely engaging with them as participants, benefits research endeavors and ensures patient priorities are addressed. Lastly, we present examples of how conducting POR leads to increased research capacity and personal growth for both patient partners and researchers.

**Conclusion:**

Building the foundation of this study through the perspectives of patient partners has provided insight into the difficulties immigrants experience when attempting to access and navigate the health care system that can only be understood through first-hand experience. Engaging patients as active partners on research teams enhances the potential of strengthened patient engagement, increased patient commitment to treatment, and leads to improved health outcomes. Furthermore, POR provides researchers, patients, and those serving the community at hand, an opportunity to learn from one another.

## Plain English Summary

Immigrants often find accessing and navigating the healthcare system difficult upon arriving in Canada. Difficulties are often due to cultural differences, language barriers, limited health literacy, and health system complexity. This article shares the experiences of our research team as we engaged in patient-oriented research (POR) to better understand the unique barriers faced by newcomers trying to access and navigate the health system after immigrating to Canada.

POR teams include researchers, patient partners, health care providers, and policymakers working together throughout each stage of the research process. This method of research embraces patient experience as expertise and provides patients an opportunity to influence decisions about their care. This research approach is aimed to improve patient outcomes by focusing on patient priorities.

Through sharing our experiences, this article defines POR, describes the creation of our research team, and shares how impactful co-creating research with patients was for our team. Lastly, we provide examples of how taking a POR approach can increase research capacity and personal growth for both patient partners and researchers.

## Background

Having practiced as a paramedic for over a decade, I (lead author LB) had witnessed many of the inequities immigrants face by not receiving culturally competent healthcare. I can recall instances that I had difficulty navigating diverse cultural beliefs and overcoming language barriers as a practitioner. I recognized that I lacked the expertise needed to embrace the varying cultural and religious beliefs I was encountering. Unfortunately, lack of cultural expertise among healthcare practitioners is common, resulting in many newcomers experiencing inequitable health services upon immigrating to Canada.

In 2019, I was appointed to a research role supported by institutional, provincial, and federal funding to build a patient-oriented research (POR) team. Based on what I had experienced as a paramedic, I aimed to reach out to newcomers and explore cultural responsiveness within prehospital care.

This article defines POR and shares the team's experiences of engaging in POR, followed by a synopsis of team member recruitment, establishment of research priorities, and building of relationships. Our team shares how joining forces with patient partners, rather than solely engaging them as participants, benefits research endeavors and ensures patient priorities are addressed. Lastly, we provide examples of how conducting POR leads to increased research capacity and personal growth for both patient partners and researchers.

## Patient-oriented research

Patient-oriented research is a method that involves patients beyond the role of participant. In 2011, the Canadian Institute of Health Research (CIHR) announced Canada’s Strategy for Patient-Oriented Research (SPOR) [1]. This strategy intended to improve patient outcomes by focusing on patient priorities. A patient engagement framework was developed by CIHR to support researchers, patients, healthcare providers, and policymakers in collaborating to address research priorities [1]. This approach resulted in patient perspectives being brought to the forefront, and recognition of the expertise patients have to offer.

Distinguished by its inclusion of patients as partners, POR focuses on patient-identified priorities, multi-disciplinary teams in partnership with relevant stakeholders, and the integration of knowledge into healthcare systems and practices [2]. Collaboration is promoted between patients, clinicians, policymakers, and researchers to optimize research design, improve participant retention, and enhance knowledge translation and the uptake of findings [2, 3]. Patients are empowered to have a fundamental role on the research team throughout the research process, breaking away from historic, paternalistic practitioner-patient relationships and evolving into an autonomous patient-centered model [4]. The contribution of clinicians and policymakers ensures the research remains practical and expedites integrating new knowledge into practice [2]. Bringing patients, clinicians, researchers, and policymakers together throughout the research process enhances the feasibility of integrating sustainable, accessible, and equitable solutions within the delivery of healthcare services [2, 3].

In the context of POR, the term ‘patient’ is an overarching term that includes individuals, informal caregivers, family members, and community or public representatives who have experience with a health issue [1, 2]. Moreover, in the context of POR, the concept of patient engagement focuses on collaboration between researchers and patient partners that empowers patients to set the research priorities, frame the research question, and perform varying components of the research depending on their areas of interest [1, 5]. Roles held by patient partners should be guided by patient partners and tailored to their unique lived experiences, interests, and strengths [6].

Also note worthy is the importance of compensating patient partners for sharing their time and expertise whenever possible. Unlike other team members who hold professional positions, patient partners often offer their time and expertise voluntarily. The patient partner rate of pay should be comparable to the pay of other professionals serving in similar roles and out-of-pocket expenses incurred due to participation (e.g., transportation, parking, childcare) be reimbursed [1, 7].

## Inviting patient partners to join forces

To begin building our POR team, I reached out to newcomers in an attempt to recruit patient partners in a variety of ways. Strategies included facilitating a public forum, assisting with a wellness clinic on a postsecondary campus, advertising at the local Newcomer Welcome Centre, and reaching out to Saskatchewan Centre for Patient-Oriented Research (SCPOR) for support across their network. When a newcomer expressed interest in joining our team, I met with them individually to learn more about them, their culture, and their experiences with the healthcare system since immigrating to Canada.

Early relationship building was beneficial as it established trust, minimizing any sense of power over or hesitancy to share, and it provided patient partners with a platform to share their priorities without being influenced by others. Recruitment efforts resulted in six individuals from various countries including the Philippines, India, United Arab Emirates, and Nigeria expressing interest in joining our POR team.

As a novice researcher who had not previously conducted POR, I was surprised by the time and effort that patient partner recruitment required. Researchers planning to engage in POR must consider time and allow for investment in this area as having more than one patient voice on the team helps to avoid single perspective bias, power over imbalances, and tokenistic participation [6, 8]. Furthermore, engaging multiple patient partners from the inception of the project encourages the generation of patient-centered approaches, questions, and methods which are likely to result in outcomes more relevant to patient priorities than otherwise would have been accomplished [5].

## Relationship building with patient partners

Considering that patient priorities often differ from clinical research priorities, I prioritized building relationships with patient partners in hopes of garnering a better understanding of what was important from a patient perspective. A focus on sharing and learning about each team member is important from the outset as it builds trust and facilitates mutual understanding among members [8]. One of the initiatives well received by our team is starting each meeting with a round table to share a personal highlight, cultural practice, or achievement. This provides team members an opportunity to share something important to them and provides an opportunity to learn about the diverse cultures our team brings to the table. Other methods employed to engage patient partners include ensuring agendas are distributed before meetings so patient partners know what to expect and providing patient partners an opportunity to touch base after meetings in case they require clarification, want to offer feedback, or feel something has not been covered adequately.

A review of studies led by the US Patient-Centered Outcomes Research Institute also found a lack of knowledge and experience in engagement paired with difficulty finding patients and stakeholders to partner with to be common challenges when engaging in POR [5]. Researchers need to consider that engaging with individuals who are not familiar with research requires more time as team members must be familiarized with research processes. Taking the time to foster an environment where patient partners are comfortable to share their thoughts openly is key in achieving meaningful participation from all team members [6].

Building and fostering strong, open relationships among POR team members is especially important when engaging in collaborative research priority setting and decision-making due to the potential of power imbalances. POR teams have potential of power imbalances because patient partners contribute alongside clinicians and policymakers. This can result in patient partners being hesitant to share openly in fear of appearing ungrateful or behaving negatively towards the professionals directly involved in their care [6]. One suggested approach is to provide patient partners an opportunity to share their perspectives anonymously to be discussed in a roundtable setting at meetings [6]. This approach creates a safe and supportive environment that encourages patient partners to contribute openly despite the potential of power imbalance.

## Research priorities driven by patient partners

Only one in five clinical studies have been found to address an issue considered a key priority by patients [9]. There have been monumental advances in clinical patient management over recent years, yet a calling for “nothing about us without us” from patients seeking broader involvement in health policy, clinical care, and research continues to grow [4, 10]. Individuals with the lived experience hold first-hand knowledge of what is needed to improve their quality of life, from overcoming troubling symptoms to enhancing care. POR holds the potential to improve health outcomes and quality of life for patients [11].

By creating a safe environment, each of our patient partners felt comfortable in sharing the challenges they have experienced in accessing and navigating the healthcare system. Many of the barriers they had encountered were rooted in the diversity of cultural norms, language barriers, limited health literacy, and the complexity of the health system. Unfortunately, these barriers often lead to resources not being utilized and hesitation in seeking medical attention which in turn leads to adverse health outcomes [12].

Mr. Alabi, a patient partner that had immigrated from Nigeria in 2013, described some of the difficulties he experienced in understanding the provincial health system upon immigrating to Canada:

It took me a long time to understand what should be done at what particular time as a newcomer to Canada. As a Permanent resident who had been in the country for more than three years, I did not know there was need to have a family doctor until my family joined me and my wife was sick. Even with that, it took us more than a month to get a family doctor and even longer to obtain health cards. (Alabi, 2021)

Mr. Xavier who immigrated from India to complete his post-secondary studies as an international student had a similar experience to Mr. Alabi when attempting to navigate the healthcare system. Mr. Xavier shares how his role as a patient partner provided him a venue to share the barriers that he faced in accessing healthcare upon immigrating to Canada:

Arriving in Canada, it was all new things around me being in a new country with lots of cultural shocks. It was a whole new world here. Like most of the newcomers to Canada, I did not have an idea about the healthcare system in Canada or how the process works here. I had no idea how to access the healthcare system. This project which has targeted to study the issues faced by immigrants in Canada was a great opportunity to share my story and issues I faced as a newcomer accessing the healthcare system (Xavier, 2021)

Barriers in accessing and navigating the healthcare system post immigration are not new as many studies have reported similar findings over the years [12–15]. Yet, an evidence-informed strategic plan to mitigate the disparities experienced by immigrants has not been determined. Despite a call to action for health services to “embrace an expanded mandate which is sensitive and respects cultural needs” dating back to the first International World Health Organization Conference on Health Promotion in 1986 [16 p.3], many policymakers have yet to directly address the health consequences associated with decisions being made or give priority to the importance of promoting culturally sensitive care that is responsive to the unique health needs of immigrant populations [13, 16].

## Stretching beyond expertise

Working with patients as research partners, rather than participants, redirected our study’s objectives to align with patient priorities rather than my priorities rooted in a personal sense of comfort and proficiency. Although redirecting the scope of the study beyond prehospital care stretched me beyond my comfort zone and incited a steep learning curve, the research evolved into something greater than originally envisioned. The opportunity to embrace POR in this manner is not always feasible. However, the uniqueness of my research role ingrained in POR allowed our team to embrace the new direction guided by our patient partners.

Recognizing personal knowledge gaps, I was deliberate when recruiting the remainder of our research team. I knew I would have to lean on varying team members heavily for their expertise throughout the project. Despite patient partners having a key role in POR, collaboration with healthcare providers and policymakers throughout the research process is crucial in ensuring outcomes reform care through improved delivery of services, programs, and policies [3, 4, 17]. Ultimately, recruitment efforts resulted in stakeholders from local newcomer and multicultural agencies as well as a nurse practitioner and a Patient and Family Centered Care Specialist from the health region joining our research team.

Our team members from local newcomer and multicultural agencies hold a vast amount of knowledge about the immigration sector and resources available to newcomers but they lack research experience. Combining our diverse areas of expertise with the lived experiences of patient partners has proven instrumental in creating our research design. Ms. Hector of the local immigration partnership expresses her sentiments regarding taking a POR approach to the research at hand by sharing:

Access to health and navigating the health system is something we have observed in our sector of serving immigrants. They [are] struggling with navigating and accessing the services they really need in a way they can understand… [Taking] the voices of all those who are experiencing the challenges and those who have been observing these challenges for years and bringing them all together [is] something really beautiful and effective. (Hector, 2020)

Our experience is a prime example of how empowering patient partners to steer the study objectives holds the potential to enhance research. Patient partners opened our eyes to the issues most important to newcomers—accessing and navigating the health care system. Working in partnership with patients, clinicians, and policymakers throughout the project helps ensure study recommendations are feasible and promotes the timely integration of knowledge into practice. Shifting the health research paradigm in a way that embraces lived experiences of patients holds great potential to improve health outcomes while increasing the research capacity of everyone involved.

## Our research project

Considering the research team’s expertise, and following extensive dialogue, the research question we developed was: How can barriers be eliminated, and supports be elevated to meet the needs of individuals immigrating from another country in accessing and navigating the healthcare system? The problem to be addressed is barriers to accessing and navigating the health system for new immigrants in a small community in a Canadian prairie province. Our research outcomes include providing recommendations to stakeholders on improving access, developing networks for new immigrants, increasing knowledge mobilization, and promoting the profile of immigrant offices as support measures for newcomers.

The methodology being employed is phenomenologically inspired. Our team aims to develop a deep understanding of the lived experiences of newcomers to Canada when attempting to access and navigate the provincial health care system. Data will be collected via semi-structured interviews. Interview questions will focus on garnering a better understanding of newcomers’ understanding of health services and experiences accessing and navigating the health system. Participant experiences surrounding culturally responsive and appropriate care will also be explored.

To date, patient partners co-created the interview guide and were provided training on how to conduct interviews if they expressed interested in engaging in data collection. Patient partner engagement will also be supported throughout data analysis and finding interpretation for translation to community programming.

## Increasing research capacity and opening the door for opportunity

POR also builds capacity within patient partners as they gain knowledge through meaningful, multidisciplinary engagement [1]. Patient partner, Mr. Singh conveys the positive impact that engaging as a patient partner has had for him:

Being a patient advisor is an incredible experience where I get to know about a lot of things related to health service. It is very beneficial for my personal knowledge because I am new over here and I do not know about the health services or how they work. As a patient advisor, I get the chance to discuss the problems I am facing with healthcare. Besides this, it's a great feeling that I am contributing something to improve the health services so that the problems we are facing will not be faced by the next generation of newcomers as well. (Singh, 2021)

Many patient partners are very passionate about research topics addressing health issues that directly impact them such as Mr. de la Torre who shares that he is proud to be a patient partner because he sees it as a venue to freely express ideas and opinions that may have a lasting favorable effect for individuals like himself (de la Torre, H., personal communication, 2021). This passion often ignites an intrinsic motivation within patient partners to build additional research competencies.

The primary goal of embracing patient partners throughout POR is to recognize their lived experiences as expertise rather than turn them into research experts [6]. At times, the opportunity to be part of a research team elicits a passion for research of which patient partners were not previously aware. The development of research competencies should not be an expectation forced on patient partners. However, accounting for existing strengths and leveraging knowledge can greatly contribute to project goals as well as have a long-lasting, positive impact [6, 17].

An example of tapping into existing strengths would be that the patient partners on our team are fluent in eight languages including English, Hindi, Bengali, Malayalam, Tagalog, Arabic, Punjabi, and Visayan, and each of them has expressed interest in providing interpretation and translation support as our team engages in data collection and analysis throughout our study. Moreover, being part of our research team has led our patient partners to engage in varying research competency development such as conducting literature searches, assisting with funding proposals, developing recruitment materials, and presenting at conferences.

Mr. Kumar is a prime example of a patient partner interested in developing additional research skills beyond sharing his lived experiences:

I am aligned toward this research because of my experience as a patient. I shared my experience as an immigrant and the difficulties faced by me to access healthcare. It took a while for me to figure out how the healthcare system works here. As a patient partner on our team, I helped explore different ways to collect the qualitative data, which could help to accomplish the research goal. I also helped prepare the interview guide for the research participants, deciding the number and type of questions together... (Kumar, 2021)

Additional opportunities that have arisen for patient partners on our team due to their engagement with research include Mr. Alabi being hired as a research associate at a neighboring university and Mr. Xavier being elected as Vice-Chair of the local Immigration Advisory Table. Although the realization of new research competencies has been beneficial for our team members, the bond we have created and the support we offer one another have proven invaluable.

## Conclusion

Building the foundation of this study through the perspectives of patient partners has provided insight into the difficulties patients experience when attempting to access and navigate the health care system. These difficulties can only be truly understood through first-hand experience. Although it requires a shift to traditional health research, POR holds great potential for high-impact outcomes. Empowering patient partners to address issues most important to them in collaboration with clinicians, policymakers and researchers promotes timely knowledge mobilization and promotes feasible integration of findings into practice. Furthermore, the research process provides researchers, patients, and those serving the community, an opportunity to learn from one another. Although this article addresses recruitment approaches and team-building strategies, it is advisable to further explore the long-term effect of leveraging POR to inform existing services and uncover how existing services could be improved or modified for such communities.

## Abbreviations

CIHR: Canadian Institute for Health Research
SCPOR: Saskatchewan Centre for Patient-Oriented Research
SPOR: Strategy for Patient-Oriented Research
POR: Patient-oriented research
